# Global guidelines, local realities: toward equitable neurocritical care, local data generation and practice patterns in low- and middle-income countries

**DOI:** 10.62675/2965-2774.20250245

**Published:** 2025-11-26

**Authors:** Viviane Cordeiro Veiga, Fernanda Chohfi Atallah, Alejandro Bruhn, Diptesh Aryal

**Affiliations:** 1 BP - A Beneficência Portuguesa de São Paulo São Paulo SP Brazil BP - A Beneficência Portuguesa de São Paulo - São Paulo (SP), Brazil.; 2 Universidade Federal de São Paulo São Paulo SP Brazil Universidade Federal de São Paulo - São Paulo (SP), Brazil.; 3 Pontificia Universidad Catolica de Chile Santiago Chile Pontificia Universidad Catolica de Chile - Santiago, Chile.; 4 Nepal Intensive Care Research Foundation Kathmandu Nepal Nepal Intensive Care Research Foundation - Kathmandu, Nepal.

Spending a night on call in a busy intensive care unit in Kathmandu or Kampala teaches you two things fast. First, the physiology of critical illness does not respect borders. Second, the information systems that support our decisions still do. While clinical guidelines are easily accessible online, the granular data that reveal how those guidelines play out on the ground are often missing. Bridging that gap is among the most important and achievable projects in global critical care.

Recent narrative work demonstrates that the weakest links in many low- and middle-income countries’ (LMICs) critical care systems are not ventilators or vasopressors but reproducible processes for collecting and using local information.^(1)^ Without timely data, it is difficult to plan staffing, justify budgets, or persuade policymakers. Yet the technical hurdles to basic data capture have never been lower.

Variation in practice underlines the urgency. The global WEAN SAFE study, enrolling nearly 6,000 ventilated patients across 50 countries, revealed striking differences in weaning strategies, extubation failure, and intensive care unit (ICU) length of stay across income groups.^(2)^ A cross-sectional survey from Malawi painted an equally vivid picture of constraints: only one ventilator bed per 1.3 million citizens and frequent stock-outs of basic consumables.^(3)^ These studies resonate because they rely on data rather than anecdotes.

Intensive care unit registries provide a workable template. Nepal's national registry now aggregates information from 20 hospitals, cataloguing case-mix, organ support, and outcomes in near real time.^(4)^ A 5-year review from Tanzania highlighted a growing burden of non-communicable disease among ICU admissions.^(5)^

Additional disease-specific cohorts, such as the VENTILOMICS survey on traumatic brain injury ventilation,^(6)^ the CREVICE protocol trial in Bolivia and Ecuador,^(7)^ and the ENIO study on acute brain injury^(8)^ illustrate how registries and cohort efforts can illuminate disparities and generate actionable knowledge in LMICs.

The coronavirus disease 2019 (COVID-19) pandemic further demonstrated the adaptability of registries. Networks already capturing routine ICU data rapidly pivoted to track ventilator allocation, steroid uptake, and vaccine status, providing early effectiveness signals in resource-limited settings.^(9)^ These datasets informed national oxygen policies and procurement decisions faster than traditional trials could.

Local data also keeps us honest about predictive tools: a global individual participant data meta-analysis reported that models derived in high-income settings showed good discrimination but poor calibration when exported to LMICs.^(10)^ There is also a broader justice angle: calls to decolonize critical-care publishing and bridge applications across income levels emphasize equitable authorship and open data sharing as part of the solution.^(11,12)^

## Bridging the translation gap from high-income country guidelines

Guidelines have undoubtedly advanced critical care. However, protocols developed in high-income countries (HICs) are not always directly applicable in LMICs, where infrastructure gaps - advanced ventilators, neuromonitoring tools, and adequately trained personnel - limit feasibility.^(13,14)^ The VENTILOMICS study - spanning 28 countries - demonstrated the potential of collaborative networks to generate geographically representative data and underscored inequities such as the limited availability of intracranial pressure monitoring and continuous electroencephalogram.^(6)^ Addressing these inequities requires both adapted recommendations and targeted investments. Table 1 outlines the International Initiative for Research recommendations across three domains -infrastructure, training, and collaboration

**Table 1:** Recommendations from the International Initiative for Research

Domain	Recommendation
Infrastructure	Funding to ensure minimum capacity for reliable data collection and storage
Training	Structured education in scientific methodology and evidence-based practice
Collaboration	Creation of regional and global research networks with equitable LMIC participation

LMIC - low- and middle-income country.

## The importance of specialized neurocritical care units

One under-discussed dimension is the role of specialized ICU care, including neurocritical care and stroke units. Even in resource-limited settings, trained personnel working in organized units can meaningfully improve outcomes for patients with severe neurological conditions. Data from LMICs suggest substantial variation in the availability of dedicated neurointensive care units, with lower access in low-income settings compared to lower-middle-income settings.^(15)^

## Evidence-based implementation strategies

Transforming evidence into action requires adaptation to local realities. The CREVICE protocol offers a model of noninvasive neuromonitoring that is feasible in settings with computed tomography access and bedside neurological monitoring, allowing quality care despite the absence of invasive intracranial pressure monitoring.^(7)^ Low-cost strategies - such as affordable ventilators, portable ultrasound, smartphone-based pupillometry, intermediate units, tele-neurocritical care, and disease-specific protocols - have demonstrated potential to reduce costs and improve outcomes.^(16,17)^

## Barriers and facilitators to implementation

**Figure 1:**
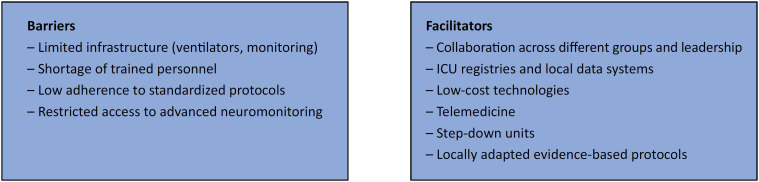
Facilitators and barriers to neurocritical care implementation in low- and middle-income countries.

Barriers include limited infrastructure, a shortage of trained personnel, and inconsistent access to essential consumables. Facilitators include collaborative research networks, open-access tools, innovative care models, and locally tailored protocols. Figure 1, summarizing these elements in two columns ("Facilitators" *versus* "Barriers"), can enhance readability and visual impact.

ICU – intensive care unit

## Equity in authorship and data sharing

Equity in authorship and data ownership is essential. Practical steps include LMIC co-leadership in multicenter projects, transparent data-sharing agreements, capacity building in study design, analysis, and writing, and prioritizing venues that privilege locally generated data.^(11,12)^

## Conclusion

Strengthening epidemiological research in LMICs is not only a matter of justice but a strategic necessity for global medicine. By expanding specialized units, investing in registries and cohort efforts, promoting equitable authorship, and adapting protocols to resource constraints, we can move toward a more inclusive, practical, and effective neurocritical care system worldwide.
